# Similar experimental study on retaining waterproof coal pillar in composite strata mining

**DOI:** 10.1038/s41598-022-05369-7

**Published:** 2022-01-25

**Authors:** Y. Q. Wang, X. Wang, J. S. Zhang, B. S. Yang, W. J. Zhu, Z. P. Wang

**Affiliations:** 1 School of Civil Engineering, Central South University, Changsha, 410075 China; 2 National Engineering Research Center of High-speed Railway Construction Technology, Central South University, Changsha, 410083 China; 3 School of Civil Engineering, Anhui Jianzhu University, Hefei, 230022 China; 4 Qingdao Hisense Real Estate Co. Ltd, Qingdao, 266071 China

**Keywords:** Hydrology, Solid Earth sciences, Engineering

## Abstract

Numerous field examples of coal seam mining show that when coal seams under confined water are mined close to faults, water inrush effects on complex mining surfaces occur. Obeying similarity rules, physical similarity models consisting of sand, lime, and plaster were used to investigate the water conducting process, along with stress and displacement measured by a combination of mechanical senor, total station, and video camera-. Comparing the physical model tests with the calculation results of elastoplastic limit equilibrium theory, the rationality of the model has been verified. Besides, a safe width of the waterproof coal pillar has been obtained. It can be demonstrated from the model observations that the coal seam in front of the mining can be divided into three areas with different characteristics of stress and displacement, namely, which are the fault-affected area, the elastic area, and the plastic yield crack area. A closed-loop water inlet and outlet pipeline composed of a water control platform that can provide stable water pressure, and water bags pre-buried in the fault was used to simulate the water conduction in the fracture zone. Integrate the development law of stress, displacement, and water conduction coming from the upper and lower walls of the fault to further determine the reasonable width of the waterproof coal pillar.

## Introduction

**Figure 1:**
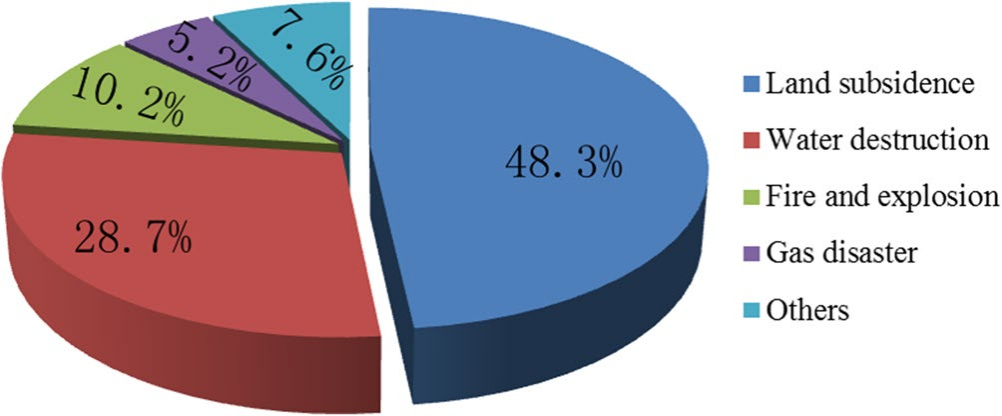
The pie chart of coal disaster proportion.

**Figure 2:**
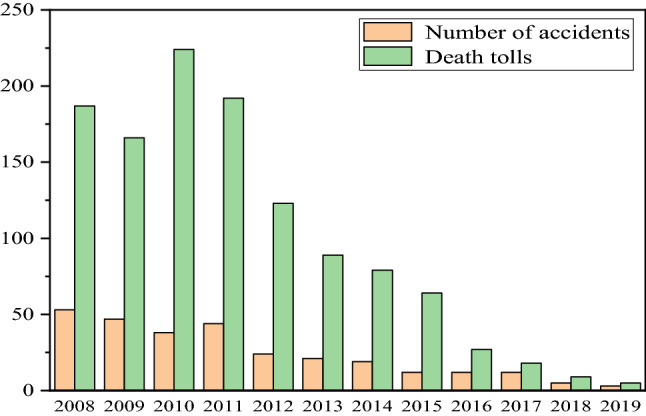
Statistical table of water inrush accidents.

There are five main types of geological disasters caused by coal seam mining: land subsidence, water resource destruction, fire and explosion disasters, gas disaster, and others, which are shown in Fig. 1^1–5^. Among the disasters, the hazards caused by coal mine water inrush are huge, as shown in Fig. 2, there were 288 water-inrush accidents, causing more than 1170 deaths in the past 12 years.

The consequences of water inrush disasters are enormous and explosive, especially when mining under the groundwater. Field examples show that when mining under aquifer, three areas with different mechanical properties can occur in the upper goaf; the caved zone, fracture zone, and the continuous deformation zone^6,7^. The water inrush channel is formed after the fracture zone broken over by the overlying aquifer in the subsidence zone, along with the confined water seeps down and flows into the mining face, which will threaten the stability of the mining face^8,9^. Thus, the primary hazards of underwater mining come from the instability of overlying strata underwater pressure, the aquifer’s flow movement, and the formation of water diversion channel^10,11^. In addition, studying the movement and deformation law of overlying strata, and the seepage-flow characteristics of water in coal seam mining has a significant theoretical guiding significance for understanding coal seam water-inrush mechanism, and the suggestions of water inrush measures.

At present, many scholars primarily focus on the overburden movement of the overlying strata, and the characteristics of water flowing within the fractured zone in underwater mining^12–14^. Adhikary obtained the water distribution of the goaf’s roof using a permeability measuring device^15^. Eberhardt^16^ studied the stress release and the stress concentration of rock caused by underground engineering excavation, and obtained the crack initiation and damage threshold. Singh conducted a new theoretical method for the classification of the water-conducting fault zones^17^. Cui summarized and analyzed the characteristics of water inrush and concluded that water inrush is closely related to roof cracks^18^. However, the overlying aquifer contains loose materials, resulting in a precarious position for the mining working force. The methods dealing with mine construction can be modified to limit this potential danger, such as dewatering, advanced drilling support, rock grouting, and the waterproof coal pillar setting^19,20^. In the above-mentioned measures, the reasonable waterproof coal pillar is considered an economical and efficient mean to prevent water inrush^21,22^. Several formulas pertaining to the strength and width of the waterproof coal pillar are proposed by scholars^23–25^. In addition, these above theories primarily focus on underwater mining, and do not concerned about confined water mining, especially coal seam mining crossing the fault^26^.

In north China, covered by a Cenozoic Quaternary thick loose aquifer, the roof and floor rocks of the coal seam have a highly water content. Generally, loose stratum breeds multiple layers of pore aquifers, is the primary stratum of water–sand inrushing. When geological structures such as faults, karsts, and fractures exist near coal seams, a water conductive channel is formed^27^. Furthermore, when the water-conductive channels are exposed during mining, water inrush accidents occur, and inrush incidents involving the fault zones are sudden and lagging^28–30^. In addition, mining further stretches the original fault structure, increasing the permeability of the fault^31,32^. These above existing theories for the thickness of the waterproof coal pillar do not consider the change of hydraulic conditions of the fault, which is a characterization quantity in reflecting water inrush.

Consequently, based on the engineering background of the Wugou’s mine 1011 work face, a similarity material test of the waterproof coal pillar for the F14 fault was utilized to investigate the stress displacement, and water conduction characteristics in coal mining. In addition, a combination of the stress, displacement, and the hydraulic condition of fault were observed to analyze the behavior of mining activities. Compared with the results of elastic–plastic limit equilibrium three-zone theory, the rationality of coal pillar width getting from the physical experimental testwas verified, which could provide some meaningful guidances for water inrush in coal mine engineering.

## Similar modeling of waterproof coal pillar width in coal mining

### Engineering background

**Figure 3:**
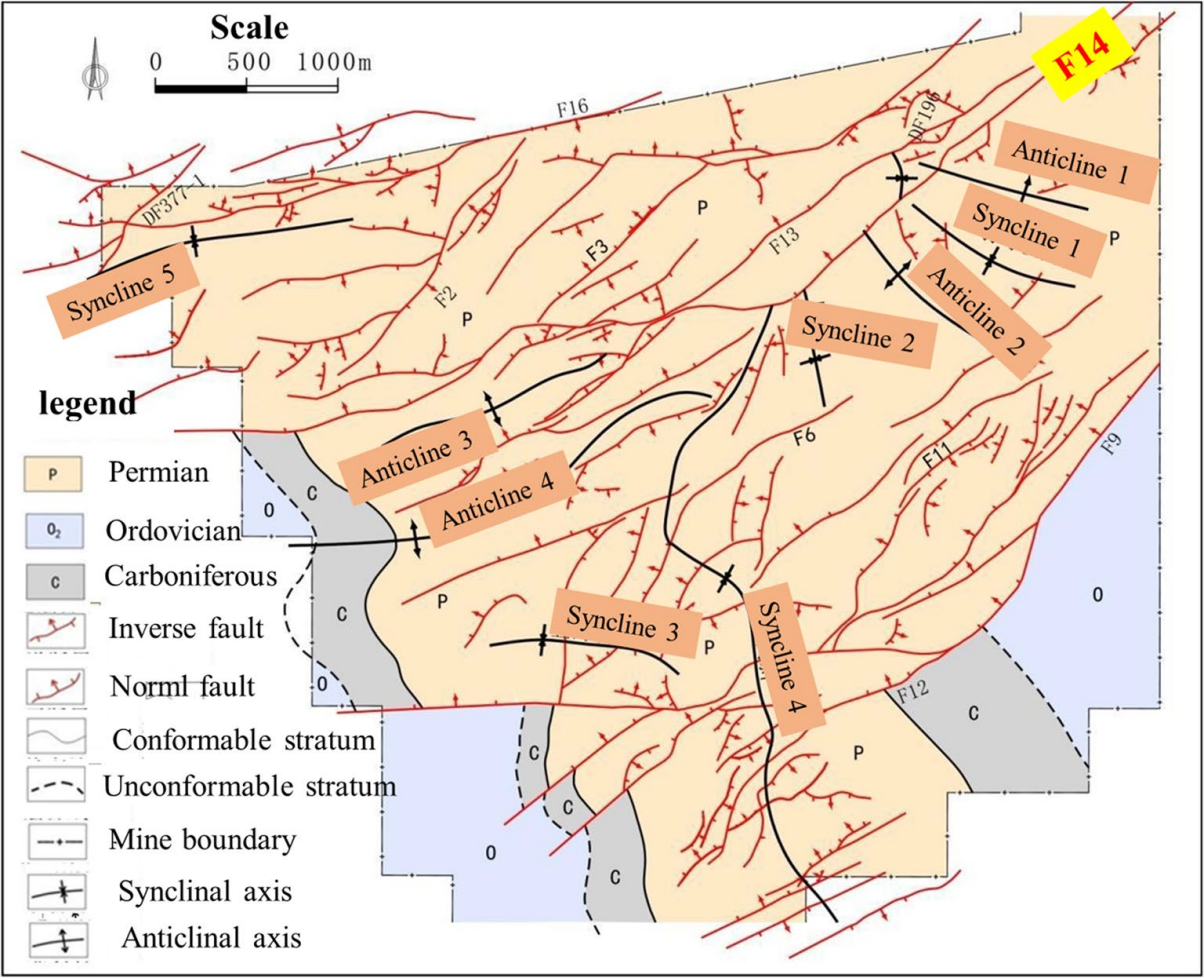
The minefield structure of Wugou mine.

**Figure 4:**
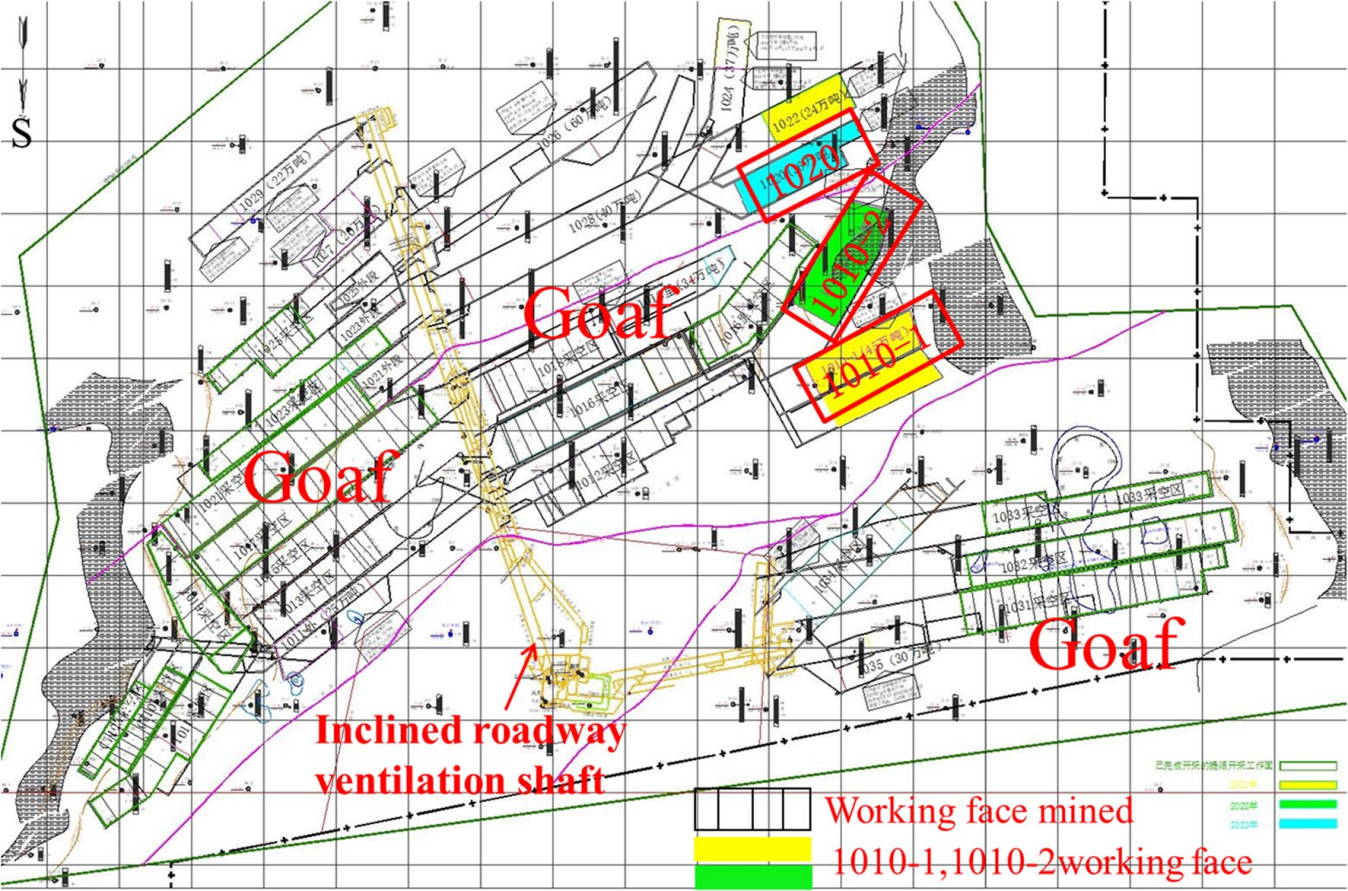
The mining surface layout and mining situation of Wugou mine.

**Figure 5:**
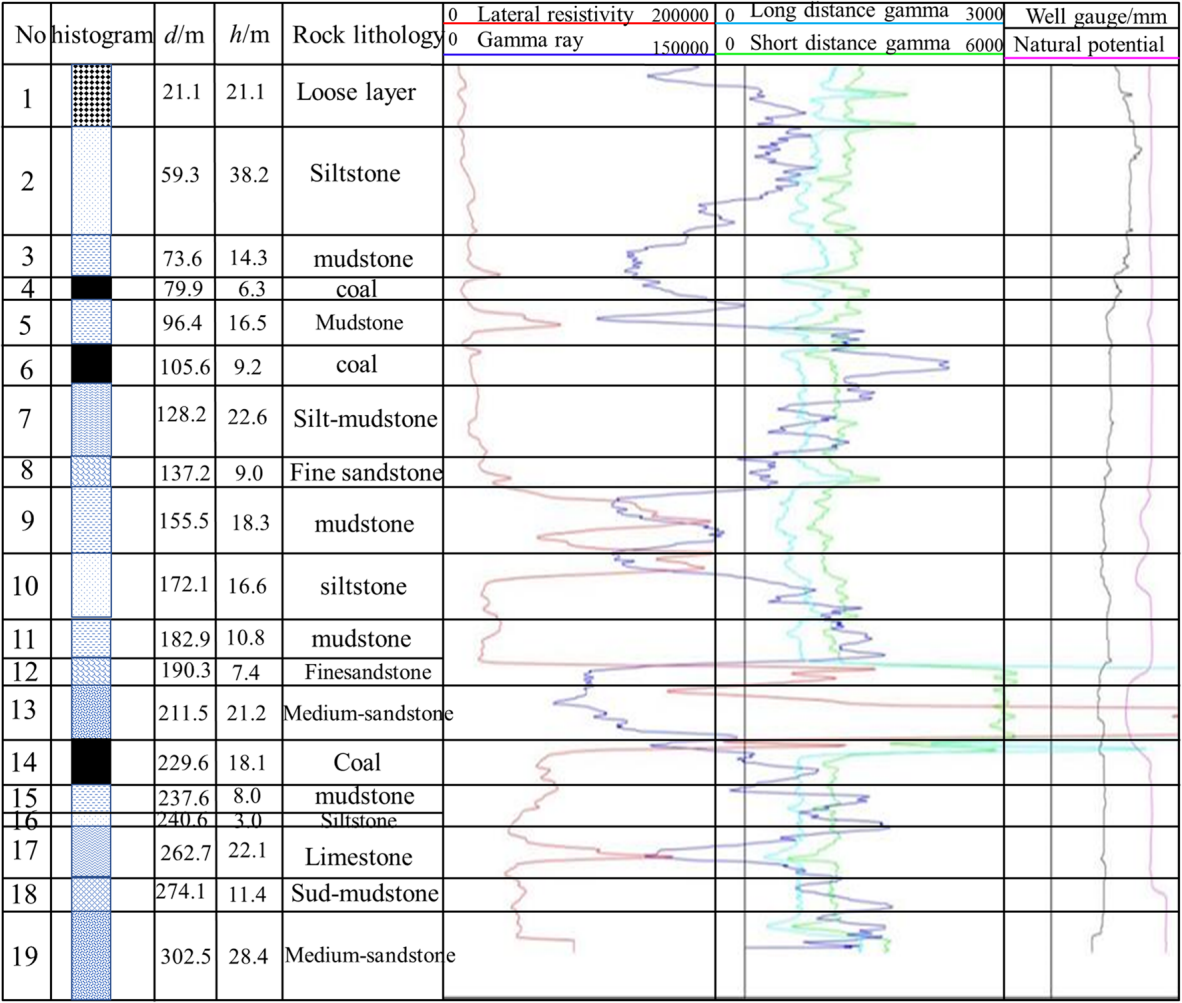
The histogram of rock formation near the 1011 work face.

Wugou Coal Mine is located in Wugou District, Huainan City, Anhui province. The 1011 working face is located in the northeast syncline area of Wugou coal mine with an average mine thickness of 5.95 m. The minefield structure and mining area arrangement of Wugou were shown in Figs. 3, and 4, respectively. The central coal seam is 10 # coal separated by a normal fault, which has an inclination angle of 70°, a drop of 20 m, and an extension length greater than 4300 m. Based on drilling data of coal seam and strata near 1011 working face, lithology histogram, buried depth, and ground stress of roof and floor of coal seam are shown in Fig. 5. The basic mechanical parameters of the rock mass in the mining area obtained through the strength test of the cylinder sample obtained by the field drilling, as shown in Tables 1, and 2, can provide a reference for the selection of rock mass parameters in similar physical tests. As seen in Table 2, the Young's modulus (*E*_*Young*_) and Poisson's ratio (*μ*_p_) were both equivalent values, which were predicted by the Kuster–Toksoz and Mori–Tanaka models^33^, and got from uniaxial compression strength test.

**Table 1:** RQD value of roof and floor strata in coal seam.

Rock type	RQD (%)	M	Rock integrity
Roof			
Loose layer	0 ~ 48/15	—	Fracture
Mudstone	30 ~ 95/71	0.025	Middle
Siltstone	48 ~ 95/72	0.045	Middle
Fine sandstone	40 ~ 100/74	0.062	Middle
Medium sandstone	60 ~ 100/78	—	Complete
Floor			
Mudstone	40 ~ 95/75	0.038	Complete
Siltstone	40 ~ 100/74	0.051	Middle
Limestone	40 ~ 95/73	0.078	Middle
Sand-mudstone	0 ~ 48/15	—	Fracture
Medium sandstone	52 ~ 95/71	0.012	Complete

**Table 2:** The mechanical properties of rock.

Lithology	*E*_Young_ (GPa)	*μ*_p_	*φ* (°)	*C* (MPa)	*R*_c_ (MPa)	*ρ*_v_ (KN m^-3^)
Loose layer	0.071	0.19	30	1.56	19.2	12.3
Siltstone	4.25	0.24	31	2.26	32	23.8
Mudstone	5.30	0.20	29	1.98	25	23
Silt-mudstone	6.78	0.23	30	2.46	33	22.5
Fine sandstone	11.54	0.18	35	4.75	44	26.5
Medium sandstone	6.85	0.22	30	8.65	33	27.9
10#coal	1.78	0.27	23.1	2.1	11	18.0
Mudstone	12.32	0.23	32	2.03	28	25.0
Siltstone	21.25	0.25	32	2.37	34	25.4
Limestone	18.56	0.23	30	4.5	35	26.6
Mud-sandstone	24.35	0.22	31	3.7	30	26.4
Medium-sandstone	23.67	0.23	31	7.86	34	27.1

### Similar theory and modeling principles

In accordance with similarity theory and field test results, the physical model was designed. Relying on the model results, the impact of mining activities was analyzed. Whittaker and Reddish stated that parameters with major influence on the mechanical behaviour of the material should be considered, including the geometric, stress, bulk density, and time similarity, along with the parameters satisfy the fundamental condition of the similarity theory as^34^:

1 $$\frac{{C_\sigma }}{{{C_\rho } \times {C_L}}} = 1$$

where *C*_L_, is the constant of geometry; *C*_σ_, is the constant of strength; *C*_ρ_, is the constant of density similarity between the model and prototype case; *L*, is length; *σ*, is compressive strength..

Strength parameters of intact rock mass can be estimated by rock mass rating system based on laboratory uniaxial test results of rock samples obtained from field drilling. Based on the theory that the average uniaxial compressive strength of natural rock mass is 28% of the indoor compressive strength of intact rock mass, the compressive strength of natural rock mass is obtained^35,36^.

**Figure 6:**
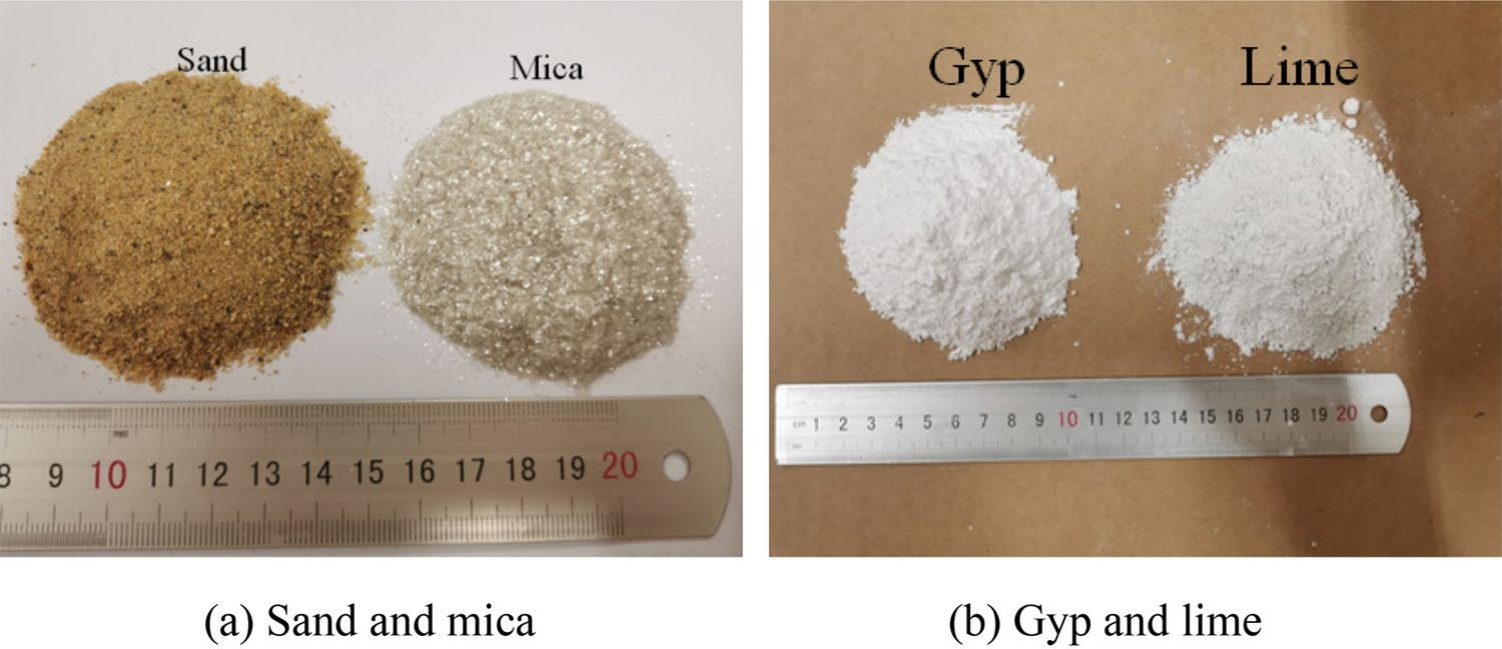
The test materials of physical model.

**Figure 7:**
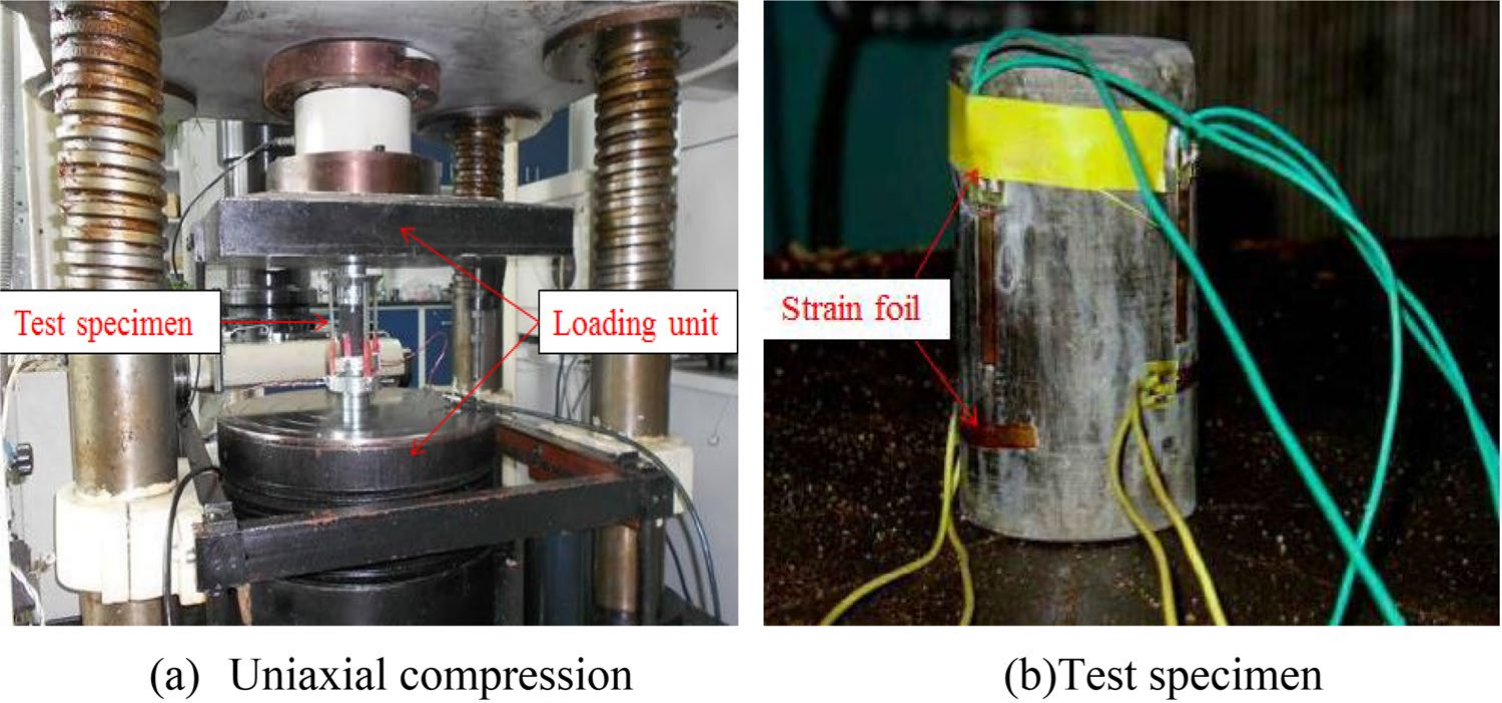
TAW-2000 rock triaxial testing apparatus and uniaxial compression test.

The aggregate is ordinary river sand, with a diameter less than 1.5 mm, and the binder is composed of lime, gypsum, and mica powder simulating the rock bedding structure. All of the materials are shown in Fig. 6. The material and water were mixed and stirred evenly to prepare a cylindrical specimen, with the influence of various materials on the compressive strength was obtained from uniaxial compression test (Fig. 7). After completing a series of uniaxial compression tests, the similarity strength parameter of the model is taken as Eq. (2). The mixture and model parameters of the prototype rock mass are shown in Table.3.

2 $${C_\sigma } = {{{\sigma_p} \mathord{\left/ {\vphantom {{\sigma_p} \sigma }} \right. \kern-\nulldelimiterspace} \sigma }_m} = 300$$

where *C*_σ_ is the constant of strength$;{\text{\;}}{\sigma_{\uprho }},{\sigma_{\text{m}}}\;$ are the compressive strength of prototype and model respectively.

**Table 3:** Physical parameters of rock similar materials.

Lithology	*Ρ*_v_ (KN m^−3^)	*μ*_m_	*C* (kPa)	*φ* (°)	*R*_c_ (MPa)	*R*_t_ (MPa)
Loose layer	7.32	0.23	23.1	28	0.064	0.21
Siltstone	14.17	0.26	69.4	32	0.107	0.65
Mudstone	13.69	0.21	71.2	31	0.083	0.62
Silt-mudstone	13.39	0.22	173	30	0.11	0.53
Fine sandstone	15.77	0.18	95	35	0.147	0.55
Siltstone	16.61	0.25	47.4	32	0.11	0.61
Coal	10.71	0.27	42	28	0.037	0.40
Mudstone	14.88	0.23	40.6	32	0.093	0.62
Siltstone	15.12	0.21	56.5	35	0.113	0.58
Limestone	15.83	0.23	90.1	30	0.117	0.70
Mud-sand stone	15.71	0.22	74.3	31	0.100	0.63
Medium sandstone	16.13	0.26	68.9	34	0.113	0.69

This experiment adopts the two-dimensional similar material simulation experiment platform of the geotechnical laboratory of Anhui Jianzhu University, which size is 3.0 m (length) × 0.4 m (width) × 1.7 m (height). Compared with the length and depth of 1011 working face, the similarity ratio of similar model is obtained as:

3 $${C_L}{ = }{{X_p} \mathord{\left/ {\vphantom {{X_p} {X_m}}} \right. \kern-\nulldelimiterspace} {X_m}} = {{Z_p} \mathord{\left/ {\vphantom {{Z_p} {Z_m}}} \right. \kern-\nulldelimiterspace} {Z_m}} = 178$$

where *C*_L_ is the constant of geometry; *X*_p_, *Z*_p_ are the length and depth dimension of working face field respectively; *X*_m_, *Z*_m_ are the length and depth dimension of similarity model, respectively.

According to Eq. (1), the density similarity ratio is *C* ${\uprho }$ = 1.68. Based on the similarity constants of model size, strength, and density of test materials, the mixing ratios of each rock layer are obtained, as shown in Tables 4, and 5.

**Table 4:** The material ratio of simulated rock strata on fault hanging wall.

No	Lithology	Thickness (cm)	Mixratio	Laying length/cm	M/(kg)			
					Sand	Caco_3_	Gyp	Water
R13	Loose layer	11.8	8:3:2	275.62	35.23	2.56	3.24	4.81
R12	Siltstone	21.4	7:5:5	271.14	43.43	3.1	3.1	4.96
R11	Mudstone	8	8:6:4	264.04	30.98	2.32	1.55	3.49
R10	Coal	3.5	8:6:4	252.49	17.78	1.33	0.89	2.00
R9	Mudstone	9	8:6:4	251.80	44.32	3.32	2.22	4.99
R8	Coal	5	8:6:4	248.34	34.97	2.62	1.75	3.93
R7	Silt-mudstone	12.5	8:6:4	246.95	31.87	2.39	1.59	3.59
R6	Fine sandstone	5	7:8:2	239.33	44.23	5.05	1.26	5.05
R5	Mudstone	10.2	8:6:4	235.87	34.59	2.59	1.73	3.89
R4	Siltstone	9.3	7:5:5	230.09	31.18	2.23	2.23	3.56
R3	Mudstone	6	8:6:4	226.28	41.15	3.09	2.06	4.63
R2	Fine sandstone	4.1	7:8:2	222.53	39.07	4.46	1.12	4.46
R1	Medium sandstone	11.9	7:7:3	219.24	40.52	4.05	1.74	4.63
M	10coal	10	8:6:4	212.31	24.91	1.87	1.25	2.80
F1	Mudstone	4.5	7:5:5	210.00	32.34	2.31	2.31	3.70
F2	Siltstone	1.7	8:6:4	207.11	26.73	2.00	1.34	3.01
F3	Limestone	12	7:5:5	205.84	32.97	2.35	2.35	3.77
F4	Sand-mud interbed	2.7	8:6:4	199.84	23.45	1.76	1.17	2.64
F5		3.5	8:6:4	198.45	23.29	1.75	1.16	2.62
F6	fine sandstone	16	7:5:5	196.14	36.25	2.59	2.59	4.14

**Table 5:** The material ratio of simulated rock stratum in fault footwall.

No	Lithology	Thickness (cm)	Mix-ratio	Laying length/cm	*M*(kg)			
					Sand	Caco_3_	Gyp	Water
F7	Siltstone	16.5	7:5:5	39.42	7.29	0.52	0.52	0.83
F8	Mudstone	4	7:6:4	41.73	5.14	0.44	0.29	0.59
F9	Coal	2	8:6:4	42.89	5.03	0.38	0.25	0.57
F10	Mudstone	6	7:6:4	46.35	8.57	0.73	0.49	0.98
F11	Coal	3.6	8:6:4	48.43	5.11	0.38	0.26	0.58
F12	Mudstone	11.5	7:6:4	55.36	10.23	0.88	0.58	1.17
F13	Fine sandstone	5	7:5:5	57.96	8.03	0.57	0.57	0.92
F14	Mudstone	5	8:6:4	60.84	8.92	0.67	0.45	1.00
F15	Siltstone	11	7:6:4	67.77	25.05	2.15	1.43	2.86
F16	Mudstone	3	8:6:4	69.16	9.74	0.73	0.49	1.10
F17	Finesandstone	2	7:6:4	70.31	8.66	0.74	0.49	0.99
F18	Medium sandstone	17	8:6:4	80.71	14.20	1.07	0.71	1.60
F19	10coal	10	7:6:4	85.61	11.21	0.96	0.64	1.28
F20	Mudstone	14	8:6:4	93.46	12.43	0.93	0.62	1.40
F21	Siltstone	16.5	7:6:4	103.86	19.19	1.65	1.10	2.19
F22	Fine sandtone	17	8:6:4	114.25	20.11	1.51	1.01	2.26

**Figure 8:**
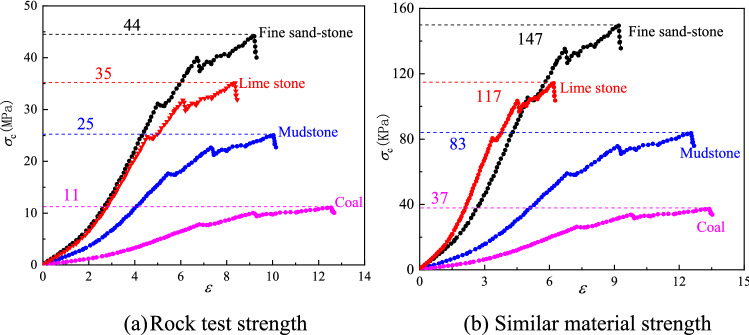
The uniaxial compression test of physical and field rock.

**Figure 9:**
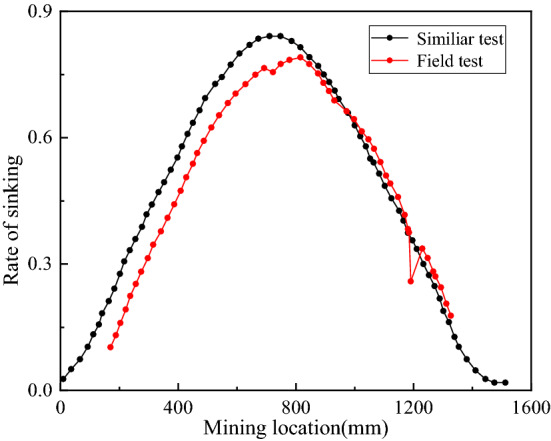
Comparison of physical model and field test results.

Comparing the uniaxial compression test results of the model sample and the on-site rock, the material strength of the model has been verified as shown in Fig. 8. Four typical rock formations were selected to verify the presented of physical model. The uniaxial compression strength ratio of the rock under the two methods of physical model and field test satisfies the Eq. (4), equalling to Eq. (2), which means the material strength of the physical model meetting the design requirements. In addition, the change trend and value of the sinking rate of field test and similar model test are consistent, as is shown in Fig. 9, along with the mining distance advancing toward the fault. Thus, it is reasonable to study coal seam mining activities using this similarity physical model.

4 $$\left\{ {\begin{array}{*{20}{c}} {{c_\sigma } = \frac{44MPa}{{147KPa}}\left( {black{\text{\;}}line} \right)} \\ {{c_\sigma } = \frac{35MPa}{{117KPa}}\left( {red{\text{\;}}line} \right)} \\ {{c_\sigma } = \frac{25MPa}{{83KPa}}\left( {blue{\text{\;}}line} \right)} \\ {{c_\sigma } = \frac{11MPa}{{37KPa}}\left( {purple{\text{\;}}line} \right)} \end{array} = 300} \right\}$$

### Physicaling modelling and construction procedure

**Figure 10:**
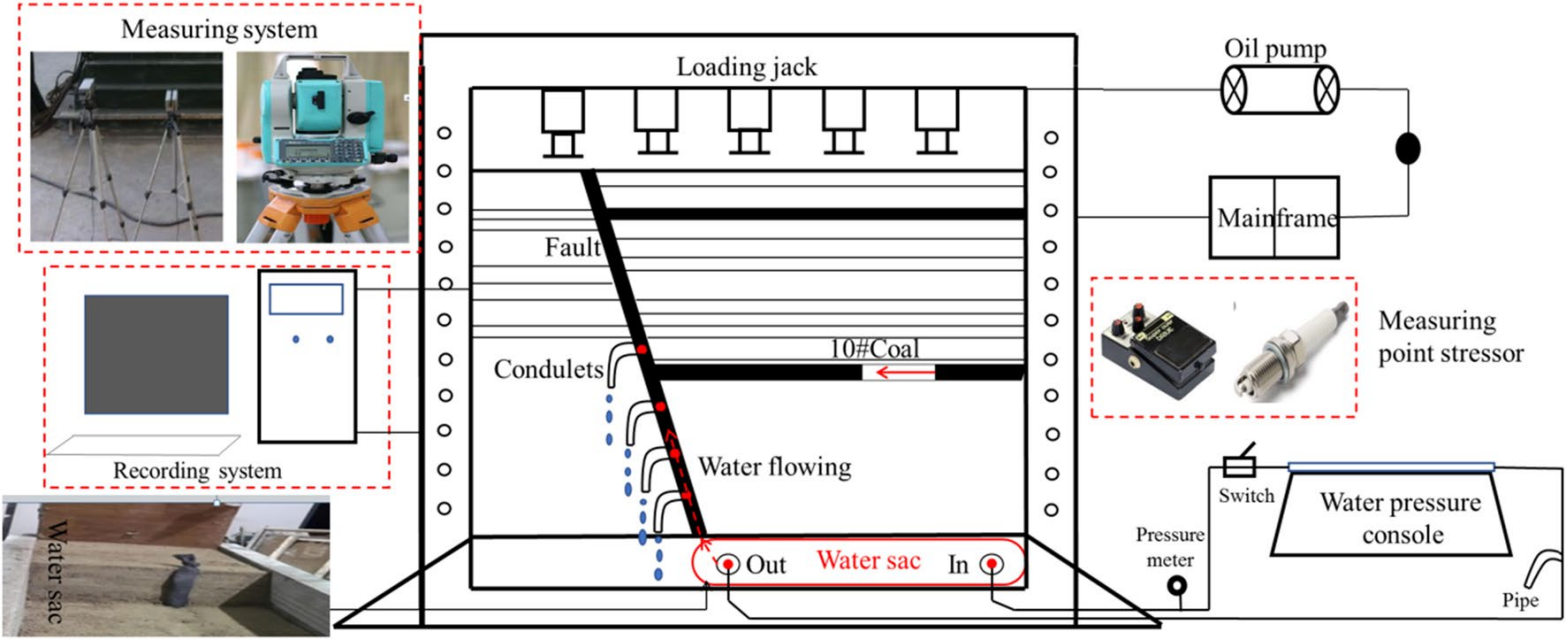
Schematic diagram of fault waterproof coal pillar loading and diversion test device.

The whole mining similar test system includes the loading system, water pressure control, recording system, measuring system, measuring point stressor, and the fault water diversion device, as shown in Fig. 10.

**Figure 11:**
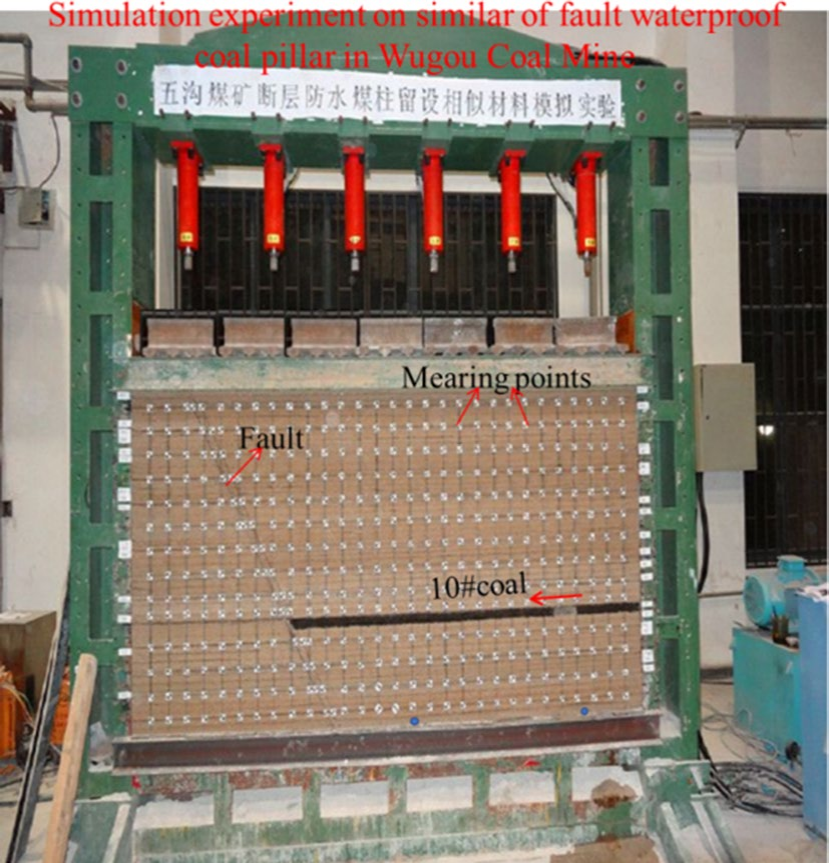
The completed model layout.

At first, the stratum boundary line and fault reference line of each rock layer were prelabeled in the steel model frame. Then, the steel was placed at the bottom of the experimental frame used as the front and rear constraints of the water sac, which was placed on the right side of the bottom in the experimental frame. The left side was filled with siltsand, along with the layed reservation of inlet and outlet of water pipe at both ends of water bag. The thin steel plate has been laid on the water sac, with paving from the bottom to the top of the fault. The well-proportioned lithologic materials were paved layer by layer from top to bottom, following the rock stratum line on the model frame. According to the layout diagram of measuring points, the stressor was installed at the corresponding position of the model, along with the connecting line connected to the measurement system. With the model paving height increasing, water-conducting bags 1, 2, 3, and 4 were installed inside the fault. After the model paved, the displacement label was affixed on the model surface, and the movement process of the displacement label was recorded by the total station and high-definition camera. The whole model was laid in accordance with the order of stirring, pouring, vibrating, leveling, and maintenance, with the completed paving model is shown in Fig. 11.

### Testing procedure and set up

#### The arrangement of measuring points

**Figure 12:**
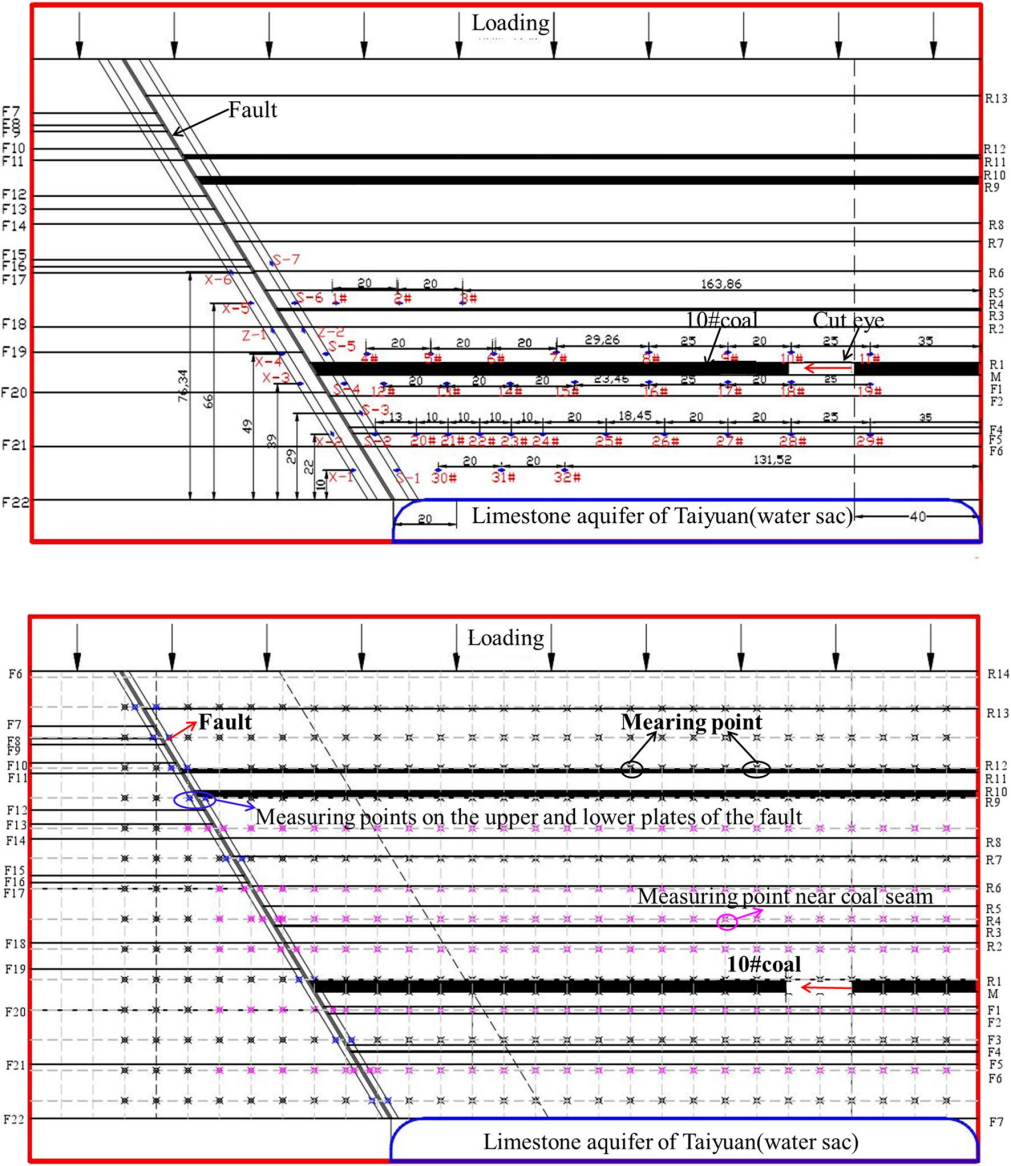
Arrangement of measuring points.

Figure 12 shows the layout of stress and displacement monitoring points. A total of 48 stress measuring points are arranged in the roof and floor of the coal seam, and the upper and lower plates of the fault. The distance and height of measuring points are shown in Fig. 12a. The displacement measuring points are evenly arranged on the coal seam strata according to the distance of 10 cm apart. In contrast, the stress measuring points of the fault are only arranged on the same horizontal position of the upper and lower plates.

#### Layout of water control system

**Figure 13:**
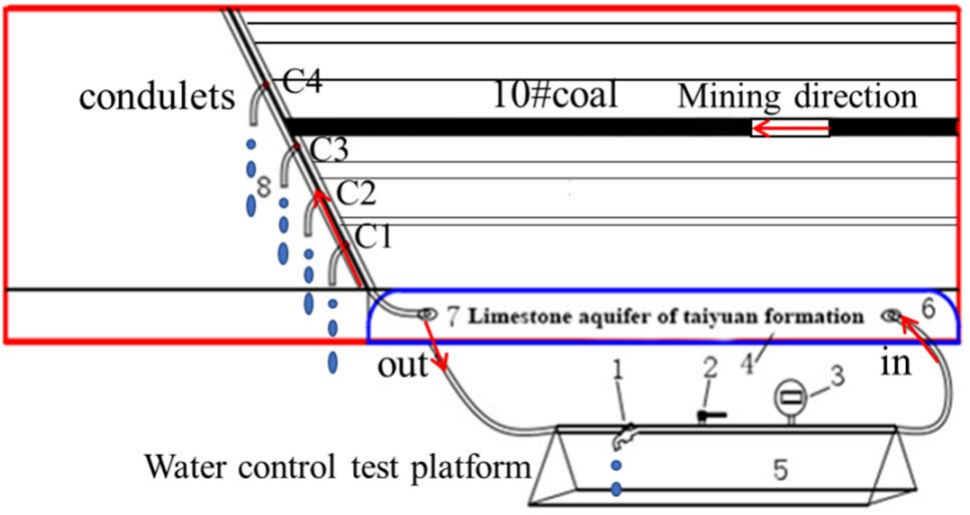
Schematic diagram of fault water diversion control system.

Figure 13 shows the simulation of the water pressure regulation within the water-conducting layer. The whole system includes water pressure control platform 5, water bag, in–out pipe, and conduit 8. The waterbag for simulating the limestone aquifer, contains outlet7 and inlet6 were arranged in the fault. The water was injected into the water-sac to keep the upper plane of the water sac equal to the bottom plane of the thin steel plate during mdel building. Before coal mining, the constant pressure water higher than the simulated water pressure is injected into the water sac to increase its water pressure, and the observation water pressure is adjusted by water gauge 3. Then the water pressure maintains at the stable value with switch 2 closed, and switch 1 opened. The above water pressure adjustment process is repeated to keep the water pressure constant throughout the mining progress. Observing and recording the flow condition, flow velocity, and water outlet time of four small pipe mouths in the fault.

## Test results and discussion

To eliminate the boundary effect of the experiment, the model open cut of 10 coal seams is 40 cm away from the right side of the experimental frame. The mining process consists of mining every 2 h, at a rate of 5 cm each time, and stops at 30 m away from fault. Approximately one and a half to 2 h was given after the upper and lower panels fully extracting to allow the deformation to be fully developed. Photographs were taken when the model stabilized following the extraction of each panel, recording the displacement and stress change of the roof and floor, as well as the lifting height of the confined water, and the drainage of the drainage pipes.

### Movement law and failure process of coal seam roof plate

**Figure 14:**
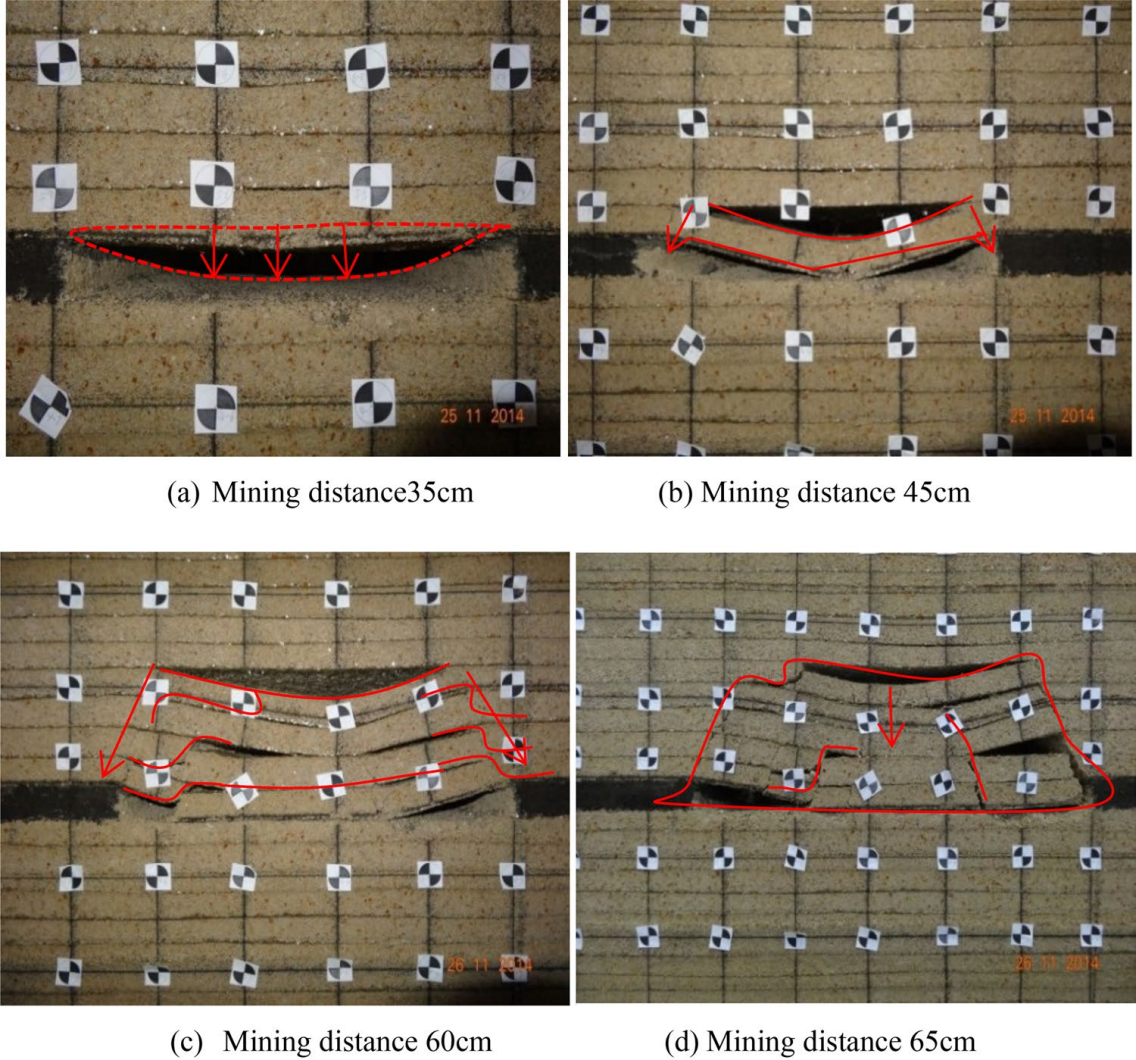
Movement process of overburden roof in coal mining advancing.

Figure 14 shows the movement process of the coal seam overburden. The movement of roof and floor can be roughly divided into four processes: the spalling–falling of immediate roof, the collapsing of primary roof, the collapsing of the main roof, and the forming of collapse zone of the main roof. The mined-out area is formed after coal seam mining, and the direct roof of coal seam is stripped (Fig. 14a). With the mining progress, the stripped range gradually extends upward, and the rock fracture cracks begin to appear. As the scope of mined-out area increases, the roof directly breaks down, sinks, and falls into the mined-out area, forming a triangular collapse area (Fig. 14b). As mining continues to move forward, two crack lines appear at both ends of the mined-out area, and the direction points to the cross-fault rock strata (Fig. 14c). The roof of the coal seam begins to collapse layer by layer along the crack line, and finally forms a trapezoidal collapse area above the mined-out area, resulting in new crack lines appear in the collapse body, as shown in curves 3, and 4, which in the collapse area constitute the main water diversion channel (Fig. 14d). The brittleness of span fracture grows obviously with the hardness of roof strata growing. Whatsmore, the fracture lines on both sides are approximately oblique and straight. These fracture of the main roof further causes bending, subsidence and separation of the overlying strata, which easily occurs in the thick and hard rock strata. The above phenomena and laws are generally consistent with the actual mine pressure observation results^37^.

### Stress variation law of the coal seam roof and floor

**Figure 15:**
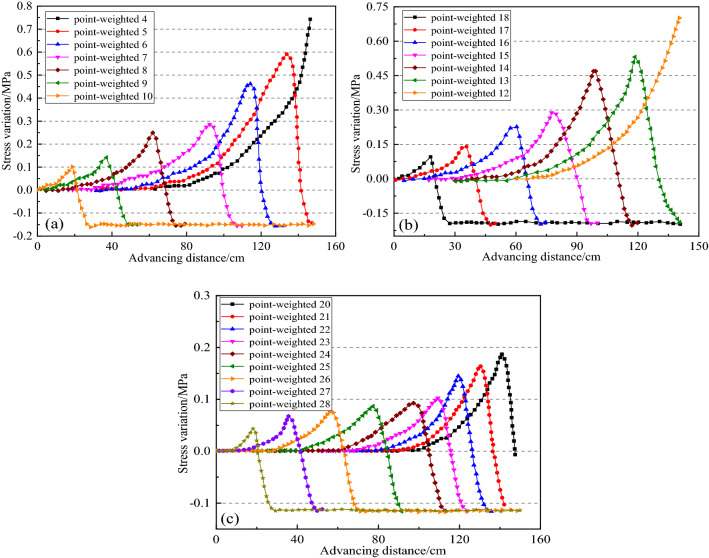
Stress variation curve of measuring points under different mining advance.

Figure 15 shows the stress variation curve of 28 measuring points around the coal seam roof and floor. It can be seen that when the mining advances to 18 cm, the stress value of measuring point 18 gradually increases to the maximum value of 0.095 MPa. However, the stress value gradually decreases as the working face passing through the measuring point. During this process, measuring point 18 becomes a goaf, floor heave occurs, and the final stress value is stable at − 0.165 MPa. The stress curves of monitoring points 17, 16, 15, 14, and 13 show the same change process. With the development of mining, the stress value of monitoring point 12 is increasing, which maximum stress variation is 0.7 MPa. The range and value of the stress increase constantly with the reduction of monitoring point number. With the mining area closer to the fault, the peak stress is greater affected by the ‘barrier’ of the fault zone, which means the coal pillar floor near the fault has undergone a relatively severe failure (see Fig. 15b).

The stress variations of measuring points 20–28 show a similar law, while the stress peak value of point 20 is 0.18 MPa, which is smaller than that of measuring point 12 due to relatively small mining disturbances far from the coal seam (see Fig. 15c). The higher concentrated stress gathers above the coal pillar, that, the closer to the working face, the more significant change of the stress at the measuring point. In addition, when mining advances to 18 cm, the stress value of measuring point 10 reaches a maximum of 0.1 MPa, and then gradually decreases as the mining face advances through point 10, the stress decreases to a negative value. Thus, the measuring point fails correspondingly. The measuring points 9–5 successively experienced the above stress-change process in turn, which further verified that the fault zone plays a “Barrier-role” in the deformation and stress propagation of rock mass during coal seam mining.

### The stress variation law of fault

**Figure 16:**
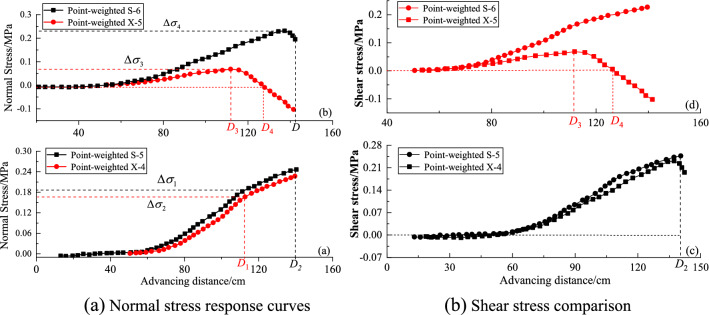
Stress variation of four measuring points of upper and lower plates under different mining advances.

Figure 16 shows the vertical stress variation curves of the four measuring points. It can be seen from Fig. 16a,b that the normal stress of the upper and lower plates of the fault gradually increases, with the mining advances to the fault. The normal stress of the hanging wall (black line) at the same level is greater than that of the bottom wall (red line). The difference value between the two plates is small and stable (0.02 MPa, Δ*σ*_1_–Δ*σ*_2_) near the coal seam, while the difference increases gradually in the far coal seam. The stress value of the lower plate point X-5 at distance *D*_3_ decreases sharply, which means that due to the influence of coal mining, the original state of the fault is destroyed, and the phenomenon of stress concentration appears. In addition, incremental shear stress far away from the seam illustrates a different profile, which has a sharp decrease, and mostly has happened after mining distance *D*_3_. This is inferred to be caused by the difference in stress release of the upper and lower walls of the fault under the influence of mining.

### The displacement law of upper and lower plates

**Figure 17:**
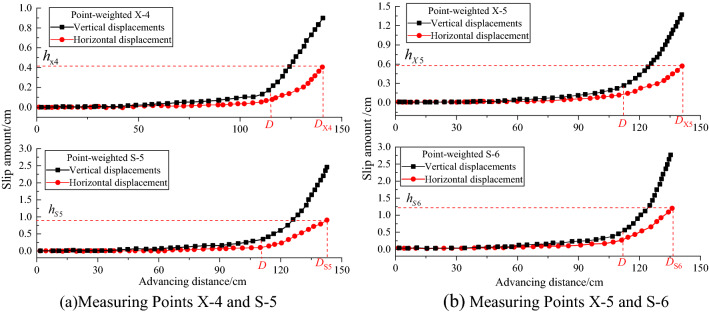
Displacement of four measuring points under different mining advances.

A total of four measuring points X-4, S-5, X-5, and S-6 on the upper and lower walls near the coal seam were used to analyze the law of displacement and settlement. Variations of the vertical and horizontal displacement curve of upper and lower wall versus the extracted coal seam distance were illustrated in Fig. 17, which growth trends are consistent, that, the trend increase slowly and steadily starting from zero to a certain distance. In addition, when the mining advanced to 110 cm (point *D* in Fig. 17), the displacement curve rises sharply until the end of mining. The maximum horizontal displacement value at the end of mining can be expressed as:

5 $${h_{S6}}\left( {1.25\,\,{\text{cm}}} \right) > {h_{S5}}\left( {0.95\,\,{\text{cm}}} \right) > {h_{X5}}\left( {0.6\,\,{\text{cm}}} \right) > {h_{X4}}\left( {0.41\,\,{\text{cm}}} \right),$$

Equation (5) demonstrates the displacement variation of the fault gradually increases, and the displacement of the hanging wall is greater than that of the foot wall along with the coal seam mining moving towards the fault. Comparing Fig. 17a and b, indicates that the vertical displacement values (black line) of the hanging wall are closed and bigger than that of horizontal displacement. This is inferred to be caused by the dfferent reactions of the upper and lower walls under the fault-influence of mining activities, which including mining distance, mining pressure, and characteristics of the fault etc.

### The water inrush law of fault

**Figure 18:**
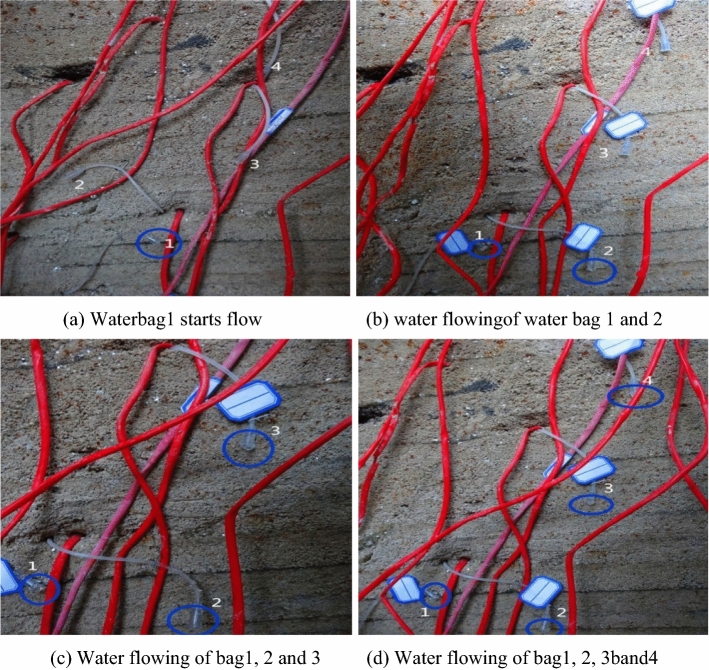
Water effluent of four water guide tubes in the fault.

The stream flow regime of the four aqueducts after the mining phase in the physical experiment was observed and recorded in Fig. 18, along with the location of upper and lower disk measuring points. It can be seen that when the mining distance is 75 cm, water bag1 starts to drip (blue line in Fig. 18a), while the water bags 2, 3, and 4 show no signs of water flowing. With mining continued, the flow rate of water bag 1 obviously increased, and when the mining reached 110 cm, water bag 2 began to drip (blue line in Fig. 18b), while the water bags 3, and 4 show no signs of water flowing. Then, the water bag 3 began to drip when mine to 130 cm (blue line in Fig. 18c), together with the increased flow rate of water bag 2, and the stable flow rate of water bag 1. In the end, water bag 4 began to drip at the mining distance 140 cm (blue line in Fig. 18c), along with the increased flow rate of water bag 3, and the stable flow rate of water bags 1 and 2. The water conduction of the four water pockets at different heights in the fault, illustrates the conduction height of the pressurized water on the floor, which also indicates the fault has been activated. After multiple-seam extraction, the original cracks in the bottom plate propagate upwards due to the depressurization effect of mining, accompanied by the generation of new cracks, which provides the possibility for the generation of water inrush channel. On the other hand, the water conductivity of the fault structure is a natural and favorable water channel, which provides convenience for the release of pressurized water.

**Figure 19:**
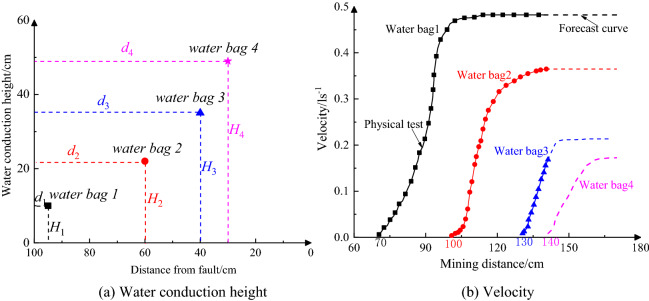
Flow velocity and water conduction height of four water bags under mining distance.

The specific water flow conditions of the four water bags are shown in Fig. 18. The flow velocity curve reflects the non-linear growth relationship between flow rate and the extracted coal seam distance, which growing trend starts from a smaller one, and then gradually increases. The maximum water velocity of pipe1 is nearly 0.5 l/s (Fig. 19b black line) with a smaller water conduction height 10 cm (Fig. 19a, black dot). Physical test results indicated that the water effuent of water bags 1, 2, and 3 before mining to 140 cm is greatly different from that of water bag 4, with the follw velocity was given by the forecast curve (purple dotted line in Fig. 19b). In contrast, the water conduction height is 48 cm, as a result of the biggest with the smallest flow velocity 0.16 l/s (purple line) of the water bag 4. In addition, the observation clearly demonstrates that, the water guiding height will greatly increase the possibility of water inrush in the coal seam face for the water guiding water bag 4 located on the top of the coal seam, which assumed that it is the critical condition of water inrush at this time, in order to ensure the safe mining of the coal seam, a conservative waterproof coal pillar width can be obtained.

## Discussion

### Calculation of coal pillar height based on similarity model

Based on the observation and analysis of the measurements from multiple-seam mining physical modeling, the calculation width of waterproof coal pillar can be obtained.

1. Roof and floor of coal seam

Comparing Fig. 15a and b, the stress measuring points14, and 6 at the roof and floor of the coal seam reach the maximum 0.5 MPa and 0.48 MPa at the mining distance 120 cm, along with the stress values of near-fault points 4, 5, 12, and 13, have exceeded the tensile strength of the coal seam, which means the roof and floor of the coal seam near the fault are damaged.

2. Displacement stress and water flow conditions of fault

To further investigate the failure propagation around the fault, stress and displacement analysis together with the water flow curve were used. The normal and shear stress curves of the footwall of the fault have a turning point at the mining position *D*_3_ (mining distance 60 cm in Fig. 15), while the stress curve of hanging wall increases sharply. Whatsmore, the horizontal and vertical displacement curves also show the same rapid increase at the position *D*_3_ (Fig. 17), which distance is used as the critical distance for safe mining.

It also has been suggested in the water conduction process of water bag 4 that the rising height of floor water reflects the possibility of water inrush at the coal mining face, which corresponding mining distance (135 cm) indicating the safety value of the waterproof coal pillar. On the other hand, the incremental flow velocity after mining coal seam illustrates changes in the stress and the water conduction state of the fault, and has mostly happened between the water bags 1, 2, and 3, which located below the mine coal area.

Considering, the mining length of 175 cm reserved from the cut hole to the fault in this model experiment (Fig. 10), due to the safety considerations, accompanying stress, displacement, and water flow conditions. The physical model gives a waterproof coal pillar thickness of 55 cm under safe mining, which can be established as:

6 $${\text{Max}}({\text{cm}})\left\{ \begin{gathered} 175 - 120\,({\text{stress,}}\,{\text{displacement}}) \hfill \\ 175 - 135\,\,({\text{velocity,}}\,\,{\text{water}}\,\,{\text{conducting}}\,\,{\text{height}}) \hfill \\ \end{gathered} \right.$$

### Elastic–plastic limit equilibrium theory considering mine pressure and water pressure

**Figure 20:**
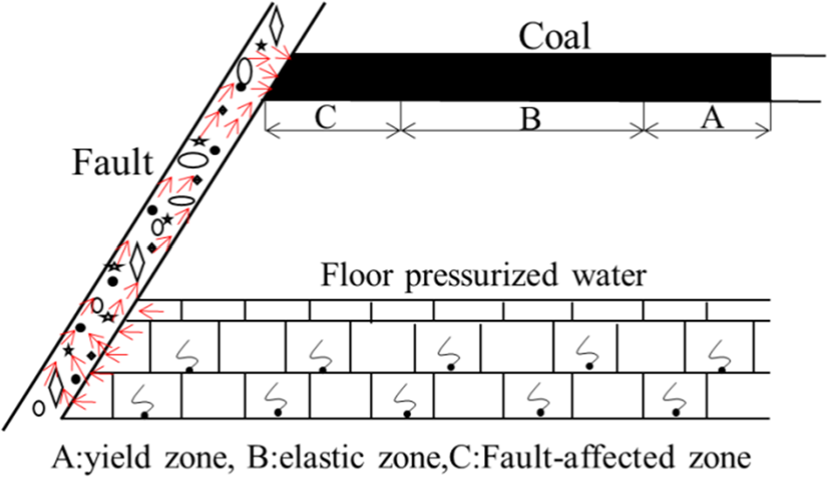
Three-zone distribution of waterproof coal pillar.

The stress redistribution of rock mass and fault under mining pressure aggravates the fracture development, fracture degree, and water conductivity of fault structure. Under the joint action of coal seam mining pressure relief and floor water pressure, the water flowing fractured zone of floor develops, and the water inrush channel forms when the water flowing fractured zone connects the boundary of fault fracture zone. In summary, the water inrush of coal seam mining when crossing the fault is the result of the joint action of mining pressure, fault development, and aquifer water pressure, as shown in Fig. 20. Considering the above three factors, the elastic–plastic limit equilibrium theory formula of waterproof coal pillar is established as:

7 $$L = {L_f} + {L_e} + {L_p}$$

where *L* is the width of waterproof coal pillar; *L*_*f*_ is the width of fault affected zone; *L*_e_ is width of elastic zone; *L*_p_ is the width of plastic zone.

#### Influence belt of fault *L*_*f*_

The width of fault affected zone is related to fault fall and lithology of upper and lower fault plates, and the relationship between them can be expressed as:

8 $${L_f} = \lambda \cdot {h^{{3 \mathord{\left/ {\vphantom {3 5}} \right. \kern-\nulldelimiterspace} 5}}},$$

where ${\uplambda }$ is lithology factor: for soft rock and coal seam, medium frequency transformer ground , hard rock stratum, the factors are 1.14, 0.76, and 0.38, respectively; *h* is fault throw.

#### Elastic zone and limit plastic equilibrium zone (*L*_e_, *L*_p_) of waterproof coal pillar

**Figure 21:**
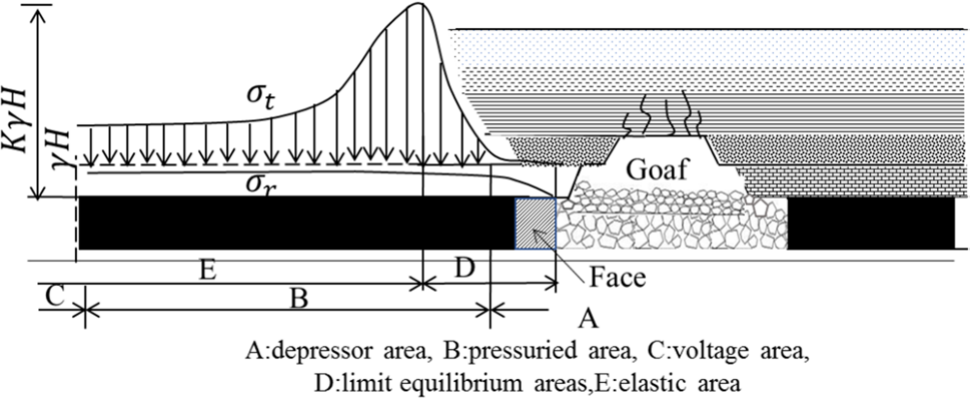
Stress distribution after coal seam mining.

After coal seam mining, the tangential stress of surrounding rock increases, first increases and then decreases along the edge of coal pillar, until the original rock stress, as shown in Fig. 21. In the whole process, the surrounding rock at the edge of the coal pillar is in a unidirectional stress state, along with a significant reduced strength got, the coal pillar is destroyed. Then the concentrated stress is transferred to the internal rock layer of the coal pillar has a one-way to three-way stress state, resulting in a gradual increased compressive strength. When the bearing capacity of the coal pillar is balanced with the pressure of the ore layer, the coal pillar retains in a stable state. According to the elastic–plastic state, the coal pillar is divided into limit equilibrium zone and elastic zone. The pressure of rock body in the limit equilibrium zone exceeds the ultimate strength of the rock stratum, resulting in the coal spalling at the edge of coal pillar and fault fracture. At this time, the water-resisting capacity of coal pillar is lost, becoming a strong permeability zone, which can be used as the residual zone of coal pillar with a low water resistance. After the peak point of tangential stress, it is an elastic zone, with good coal seam integrity and strong water-resisting ability, which is the main water-resisting rock mass. The sum of coal pillar length in limit equilibrium zone and elastic zone are the required length of waterproof coal pillar.

1. Width of elastic zone

**Figure 22:**
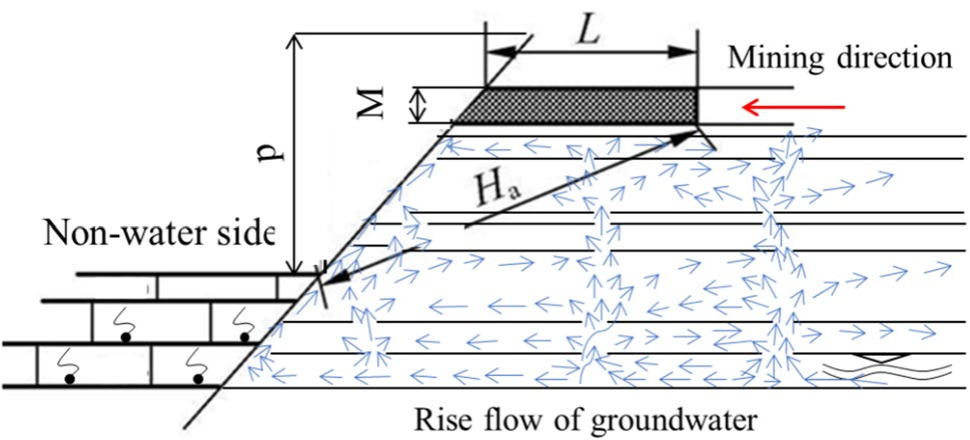
The effect of floor water rise on coal seam influence zone.

The elastic zone of waterproof coal pillar plays a major role in water insulation. According to the simple supported beam model, as shown in Fig. 22, the thickness formula of the elastic zone is expressed as:

9 $${L_e} = 0.5KM\sqrt {{{3p} \mathord{\left/ {\vphantom {{3p} {K_t}}} \right. \kern-\nulldelimiterspace} {K_t}}} ,$$

where *K* is safety factor, 2–5; *M* is height or thickness of coal seam mining, 5.95 m; *p* is water pressure, 2.6 MPa; *K*_t_ the tensile strength of coal seam,0.5 MPa.

2. Width of plastic zone

**Figure 23:**
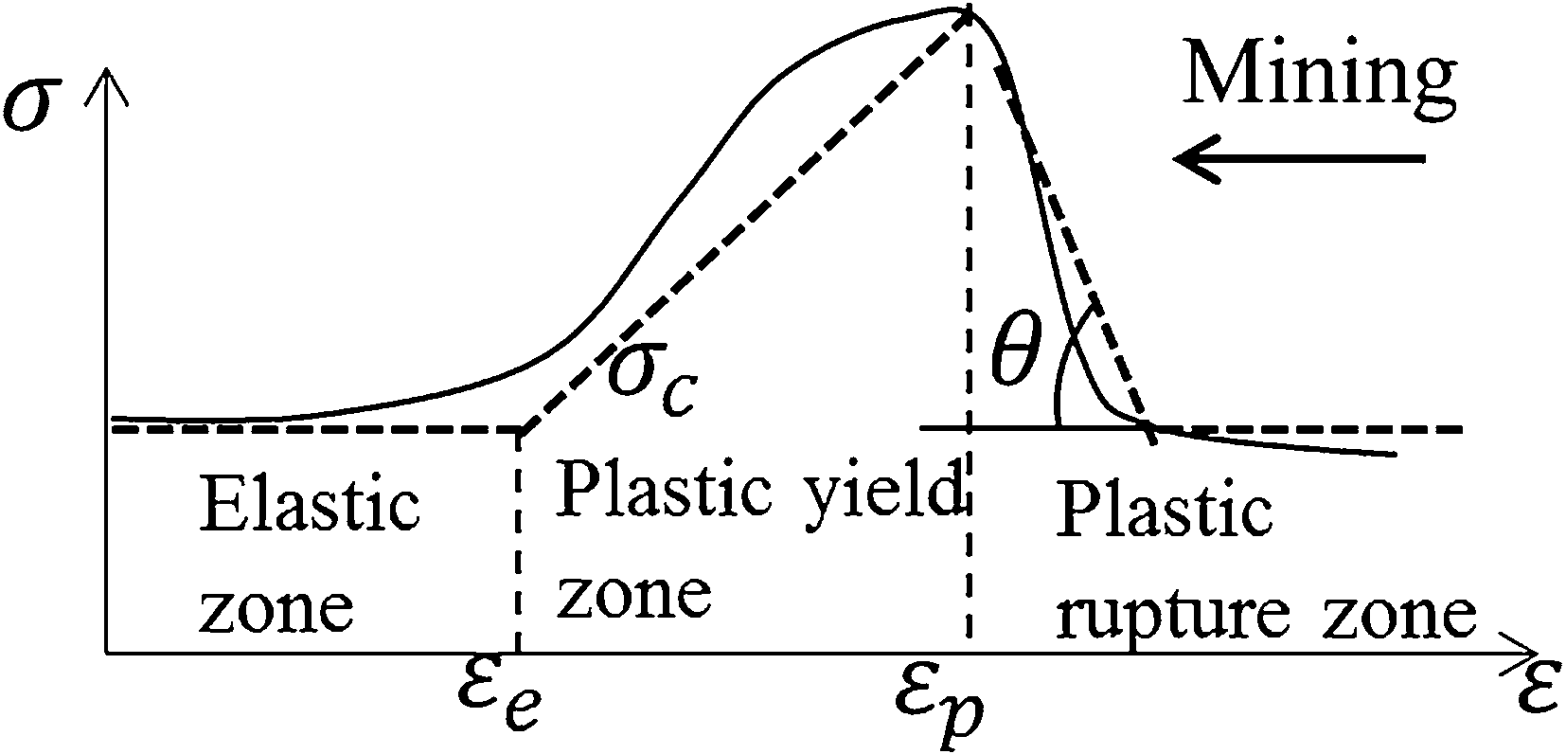
Stress–strain curve of coal seam.

Coal seam is regarded as an ideal elastic–plastic material. Based on the experimental results of many scholars, the stress–strain curve of coal seam mining is divided into three parts: the plastic fracture zone of the sharp rise curve section, the plastic yield zone of the sharp decline section, and the elastic zone of the stress stable section, as shown in Fig. 23.

**Figure 24:**
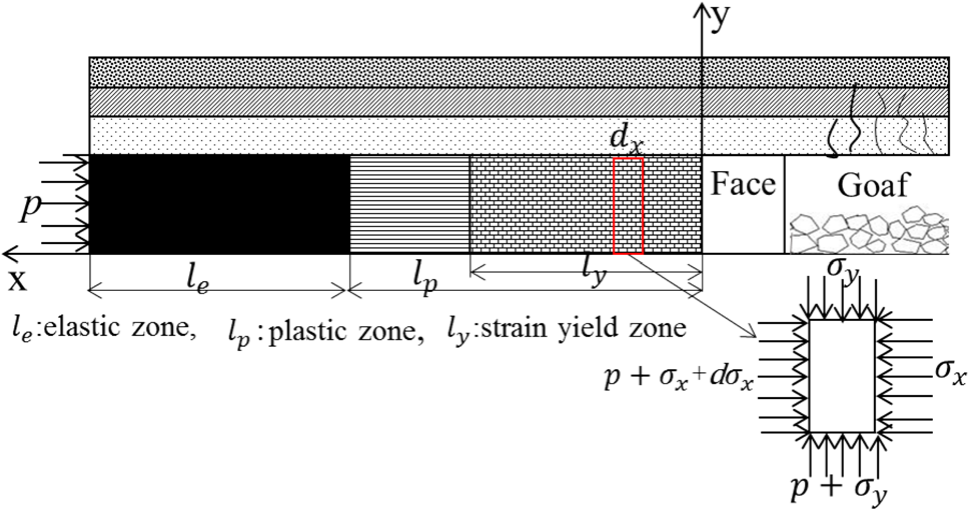
Stress analysis model of plastic zone of waterproof coal pillar.

Figure 24 shows the stress state of coal pillar in plastic zone under critical state of water inrush. Assuming that the lateral action of coal seam floor and mining front has water pressure at this time, the micro-element is taken for stress analysis, and the stress is balanced in the vertical and horizontal directions. The stress analysis of the element is carried out as^21^:

10 $$\left\{ \begin{gathered} M{\sigma_x} + 2{\sigma_y}\tan \varphi dx - M(p + {\sigma_x} + d{\sigma_x}) = 0 \hfill \\ M(p + d{\sigma_x}) - 2({\sigma_y} + p)\tan \varphi dx = 0. \hfill \\ \end{gathered} \right.$$

In the plastic yield stage, the stress strength of the coal seam meets the Mohr–Coulomb criterion, while in the plastic fracture zone, the cohesion of the coal seam decreases to the residual strength, as is shown in Eq. (11):

11 $${\sigma_1} = {\sigma ^{\prime}_c} + {k_p}{\sigma_3}$$

where ${\sigma ^{\prime}_c}$ is the yield strength of coal seam under uniaxial compression test.

Considering the similarity of the friction state between the roof and floor of the coal seam in the field test and the laboratory test, it can be reasonably assumed that the stress state under the two conditions is the same, as shown in Eq. (12):

12 $${\sigma_y} = {\sigma ^{\prime}_c} + {k_p}{\sigma_x}$$

The stress formula of the plastic yield zone is shown in Eq. (13):

13 $$\left\{ \begin{gathered} {\sigma_x} = \frac{{{{\sigma ^{\prime}}_c}}}{{k_p}}({e^{Dx}} - 1) \hfill \\ {\sigma_y} = {{\sigma ^{\prime}}_c}{e^{Dx}} \hfill \\ D = \frac{{2{k_p}\tan \varphi }}{mp} \hfill \\ {k_p} = \frac{1 + \sin \varphi }{{1 - \sin \varphi }} \hfill \\ \end{gathered} \right.$$

In the plastic yielding stage, *x* = *x*_p_,${\text{\;}}{\sigma_y} = K\gamma H$, the length of plastic zone is shown as:

14 $${l_p} = \frac{1}{D}\ln \left\{ {\frac{1}{{{{\sigma ^{\prime}}_c}}}\left[ {K\gamma H - D\left( {{e^{{{^{({\sigma_c} - {{\sigma ^{\prime}}_c})}} \mathord{\left/ {\vphantom {{^{({\sigma_c} - {{\sigma ^{\prime}}_c})}} D}} \right. \kern-\nulldelimiterspace} D}}} - 1} \right)} \right]} \right\}$$

where *m* is coal seam thickness; *K* is the underground pressure concentration factor at the elastoplastic junction; *γ* is the volume weight of the overlying rock of the coal seam; *H* is the mining depth; *p* is the water pressure of floor;${\text{\;}}\varphi$ is the internal friction angle of coal seam. All parameters and calculation results of elastoplastic limit equilibrium theory are shown in Table 6.

**Table 6:** Parameter values and calculation results of elastoplastic limit equilibrium theory.

Width	Parameters								Results
*L*_*f*_	${\uplambda }$ 0.76–1.14			*h*/m 20					4.59–6.88
*L*_*e*_	*k* 2–5		*m*/m 5.95		*p/*MPa 2.6		*k*_t_/MPa 0.51		23.5–58.7
*L*_*p*_	*k* 3	*γ/*N m^3^ 0.2	*H*/m 302.5	$\varphi$ 23.1	*m*/m 5.95	${\sigma_c}$/MPa 15.5	${\sigma ^{\prime}_c}$/MPa 12.09		21.57
*L*								49.56–87.15	

Comparing the calculation results between theoretical method and physical model, the rationality of the width of the waterproof coal pillar obtained by the physical model can be verified as:

15 $$55 \in \left[ {49.56\;87.15} \right]$$

Physical model method is an effective way in prediction of waterproof coal pillar width. However, the limited available information on rock mass strength parameters, and the difficulties to realize the simulation of the mine field model restrict the application. Furthermore, physical models are heavily field surface survey information dependant. With coal pillar cases in coal seam mining being insufficient at present, it is necessary to use a physical similarity model from other coalfields for verifying the adaptability and rationality of the physical model. The physical modeling technique presented in this paper is considered to be practical and reasonable method for investigating coal pillar characteristics in coal mining.

## Conclusions

Mining on confined water in composite strata often results in a greater possibility of water inrush in the mining surface, especially when the coal seam penetrates through the fault structure zone makes it difficult to be accurately determined only using traditional theoretical methods. Along with the correct understanding of the water inrush mechanism, the cause and setting up of waterproof coal pillars are needed. Physical modeling techniques were employed in this study to investigate the process of coal seam mining.

1. Vertical displacement of the overlying strata in the goaf increases with the advancement of the mining face and transfers from lower to the upper strata, along with cracks from caving and fracture zones. When the mining seam faces toward the fault, the stress concentration near the fault increases accordingly, and the stress value of the plastic zone in the roof and floor of the coal seam at different position increases in different degrees, with the maximum value ranging from 0.2 to 0.8 MPa.
2. The maximum water-conducting height of the fault simulated by four waterbags is approaching 50 cm, which value is much higher than that of coal seam mining height, and increases the possibility of water inrush. The flow rate of water flowing increases nonlinearly with decreasing distances between the mining face and the fault, and the average value of the flow velocity ranges from 0.1 to 0.25 m/s. Affected by the mining activities, the original cracks gradually develop and expand, leading to the increased water conductivity. Considering the "barrier" of the fault zone, the stress concentration in the vertical direction of the coal pillar occurs, as a result of mining distance gradually approaching the fault. In the fault structure zone, the confined water rises along the fault structure zone, which significantly increases the water-inrush possibility of the working face. Thus, leaving reasonable waterproof coal pillars can effectively block the water inrush path and ensure safe mining.
3. The physical model method is effective way to study on waterproof coal pillar retaining, with a reasonable width of 55 cm given by comparing with the theoretical results of three-zone elastoplastic limit equilibrium. Based on the similarity ratio of the physical model, the reasonable coal pillar width in the 1101 working face of Wugou Coal Mine is 55 m, which is safe and conservative for coal seam mining, and reasonable to be applied in engineering project.

Comprehensive application of monitoring devices, similar models of loading devices and water guiding devices have been demonstrated to be effective ways for studying the safety thickness of coal seam mining against water inrush. The model research results can provide insight into the study of the width of the waterproof coal pillar to further develop an effective waterproof coal pillar retention method. The accuracy and adaptability of the physical model rely more on geological survey information, and the determination method of rock mechanical parameters. Thus, the physical model test seeking more effective comprehensive geological information, and rock mass mechanical parameter simulation method, has become the top priority of further research.
